# The Organisation of the Medical Profession

**Published:** 1907-03-16

**Authors:** 


### The Organisation of the Medical Profession.

A remarkable series of reports from the Standing Committees of the British Medical Association has just been issued. The reports, which contain a number of recommendations, show that the medical profession is undergoing a silent but none the less important process of organisation, and one, too, which bids fair in a short time to revolutionise the conditions under which medical practice is conducted. The movement, broadly speaking, is democratic. Thus in connection with Provident Dispensaries the two principles which are considered of vital importance are that "the profession shall be able to exercise complete control over the conditions of their service, and that all practitioners, who so desire, shall have the opportunity of sharing in the work." Two reports have been issued; one concerned with the relation of the profession to its own members, the other dealing with its relations to the poorer members of the community.

The first report treats of the ethical aspects of medical consultation. It is pointed out that "the professional knowledge of the practitioner in attendance makes it highly expedient that he should, as a rule, be asked to select the consultant; but if the patient or his friends insist upon making the choice this should not be resisted by the practitioner unless the difficulty for which the consultation is required is of a kind calling for special skill which the proposed consultant does not possess, or the proposed consultant is one whom the practitioner is obliged to refuse to meet upon certain ethical grounds" which are afterwards explained in detail. Some interesting recommendations are made as to the way to evade the meeting of an unacceptable consultant. The practitioner in attendance is to reply to such a proposition that "for reasons personal to himself he cannot meet the practitioner in question"—a reply which strongly reminds one of the advice given by John of Arderne—a master in medical diplomacy—in 1348, who says: "If there be made speech to him of any leech, neither set he him at nought nor praise him too much nor commend him, but thus may he answer courteously, I have not any knowledge of him nor have I heard anything of him but what is good and honest; and of this shall honour and thanks of each party increase and multiply; after this saying Honour is in the honourant and not in the honoured." The report ends with an expression of opinion that there may be advantage both to the profession and the public in the formal recognition of a distinct class of medical practitioners who might be specially designated "Consultants," the term being defined as meaning "practitioners who have adopted the rule of seeing those patients only who are referred to them by other practitioners already in attendance and who do not themselves act as the regular attendants in any cases."

The second report deals with the relation of the medical profession to contract medical practice under the headings of the public medical services and provident dispensaries. Here the recommendations are framed with the avowed object of bringing contract medical practice under the direct control of the medical profession and of throwing it open to all practitioners in the district who choose to participate. The same spirit of democracy pervades the recommendations about hospitals. It is desired, very properly, to discountenance, as far as possible, the repeated treatment of out-patients, and eventually to replace this system by medical consultations where a patient is sent to a hospital by his medical attendant for advice upon some definite point of difficulty which has occurred in the course of the illness for which he is being treated.

The adoption of the recommendations contained in these reports would do much to bring the members of the profession into line; it would also materially benefit the poorer classes, and would utilise the resources of the hospitals to better purpose than at present. But the details would have to be carefully watched in the working, for no one would wish medicine, with its great traditions as a profession, to become a mere trade governed by trade union rules and managed by caucus meetings. It would be a matter for regret if the movement took such a direction. It will not do so at present, because the rank and file of the profession are not yet educated to a knowledge of their strength; but when this knowledge is gained, much care will have to be taken by those in power at the time, for with them will lie the making or marring of a goodly heritage.

